# Theoretical potential for endometrial cancer prevention through primary risk factor modification: Estimates from the EPIC cohort

**DOI:** 10.1002/ijc.32901

**Published:** 2020-02-18

**Authors:** Renée T. Fortner, Anika Hüsing, Laure Dossus, Anne Tjønneland, Kim Overvad, Christina C. Dahm, Patrick Arveux, Agnès Fournier, Marina Kvaskoff, Matthias B. Schulze, Manuela Bergmann, Antonia Trichopoulou, Anna Karakatsani, Carlo La Vecchia, Giovanna Masala, Valeria Pala, Amalia Mattiello, Rosario Tumino, Fulvio Ricceri, Carla H. van Gils, Evelyn M. Monninkhof, Catalina Bonet, José Ramón Quirós, Maria‐Jose Sanchez, Daniel‐Ángel Rodríguez‐Palacios, Aurelio B Gurrea, Pilar Amiano, Naomi E. Allen, Ruth C. Travis, Marc J. Gunter, Vivian Viallon, Elisabete Weiderpass, Elio Riboli, Rudolf Kaaks

**Affiliations:** ^1^ Division of Cancer Epidemiology German Cancer Research Center (DKFZ) Heidelberg Germany; ^2^ Nutrition and Metabolism Section International Agency for Research on Cancer (IARC) Lyon France; ^3^ Department of Public Health University of Copenhagen Copenhagen Denmark; ^4^ Danish Cancer Society Research Center, Diet, Genes and Environment Copenhagen Denmark; ^5^ Department of Public Health Aarhus University Aarhus Denmark; ^6^ Department of Cardiology Aalborg University Hospital Aalborg Denmark; ^7^ CESP, Faculté de Médecine, Université Paris‐Sud, UVSQ, INSERM Université Paris‐Saclay Villejuif France; ^8^ Gustave Roussy Villejuif France; ^9^ Breast and Gynaecologic Cancer Registry of Côte d'Or, Georges‐François Leclerc Cancer Centre UNICANCER Dijon France; ^10^ Department of Molecular Epidemiology German Institute of Human Nutrition Potsdam‐Rehbruecke Nuthetal Germany; ^11^ Institute of Nutrition Science University of Potsdam Potsdam Germany; ^12^ Human Study Center German Institute of Human Nutrition Potsdam‐Rehbruecke Nuthetal Germany; ^13^ Hellenic Health Foundation Athens Greece; ^14^ 2nd Pulmonary Medicine Department, School of Medicine National and Kapodistrian University of Athens, “ATTIKON” University Hospital Haidari Greece; ^15^ Department of Clinical Sciences and Community Health Università Degli Studi di Milano Milan Italy; ^16^ Cancer Risk Factors and Life‐Style Epidemiology Unit Institute for Cancer Research, Prevention and Clinical Network – ISPRO Florence Italy; ^17^ Epidemiology and Prevention Unit Fondazione IRCCS Istituto Nazionale dei Tumori di Milano Milan Italy; ^18^ Dipartimento Di Medicina Clinica e Chirurgia Federico II University Naples Italy; ^19^ Cancer Registry and Histopathology Department Provincial Health Authority (ASP) Ragusa Italy; ^20^ Department of Clinical and Biological Sciences University of Turin Turin Italy; ^21^ Unit of Epidemiology Regional Health Service ASL TO3 Turin Italy; ^22^ Julius Center for Health Sciences and Primary Care, University Medical Center Utrecht Utrecht University Utrecht The Netherlands; ^23^ Catalan Institute of Oncology (ICO‐IDIBELL)|Cancer Epidemiology Research Program, Unit of Nutrition and Cancer L'Hospitalet de Llobregat Barcelona Spain; ^24^ Public Health Directorate Asturias Spain; ^25^ Andalusian School of Public Health (EASP) Granada Spain; ^26^ Instituto de Investigación Biosanitaria de Granada (ibs.GRANADA) Granada Spain; ^27^ CIBER Epidemiology and Public Health (CIBERESP) Madrid Spain; ^28^ Universidad de Granada Granada Spain; ^29^ Department of Epidemiology Regional Health Council Madrid Spain; ^30^ Hospital Universitario Reina Sofía Murcia Spain; ^31^ Navarra Public Health Institute Pamplona Spain; ^32^ Navarra Institute for Health Research (IdiSNA) Pamplona Spain; ^33^ Public Health Division of Gipuzkoa Biodonostia Health Research Institute, Ministry of Health of the Basque Government San Sebastian Spain; ^34^ Nuffield Department of Population Health University of Oxford Oxford United Kingdom; ^35^ International Agency for Research on Cancer (IARC) Lyon France; ^36^ Department of Epidemiology and Biostatistics, School of Public Health Imperial College London London United Kingdom

**Keywords:** endometrial cancer, primary prevention, risk factors

## Abstract

Endometrial cancer (EC) incidence rates vary ~10‐fold worldwide, in part due to variation in EC risk factor profiles. Using an EC risk model previously developed in the European EPIC cohort, we evaluated the prevention potential of modified EC risk factor patterns and whether differences in EC incidence between a European population and low‐risk countries can be explained by differences in these patterns. Predicted EC incidence rates were estimated over 10 years of follow‐up for the cohort before and after modifying risk factor profiles. Risk factors considered were: body mass index (BMI, kg/m^2^), use of postmenopausal hormone therapy (HT) and oral contraceptives (OC) (potentially modifiable); and, parity, ages at first birth, menarche and menopause (environmentally conditioned, but not readily modifiable). Modeled alterations in BMI (to all ≤23 kg/m^2^) and HT use (to all non‐HT users) profiles resulted in a 30% reduction in predicted EC incidence rates; individually, longer duration of OC use (to all ≥10 years) resulted in a 42.5% reduction. Modeled changes in not readily modifiable exposures (i.e., those not contributing to prevention potential) resulted in ≤24.6% reduction in predicted EC incidence. Women in the lowest decile of a risk score based on the evaluated exposures had risk similar to a low risk countries; however, this was driven by relatively long use of OCs (median = 23 years). Our findings support avoidance of overweight BMI and of HT use as prevention strategies for EC in a European population; OC use must be considered in the context of benefits and risks.

## Abstract 2

What's new?

Endometrial cancer rates vary considerably around the world, with incidence rates higher in Europe and North America than in parts of Asia and Africa. Here, the authors investigated how much of the risk disparity arises from modifiable factors, and how much modifying these factors could reduce cancer incidence. The 10% of European women with lowest risk had similar incidence to women in low‐risk countries, they found. Their model predicted that in European women, maintaining BMI below 23 kg/m^2^ and avoiding postmenopausal hormone use could reduce risk by 30%. Long‐term use of oral contraceptives could reduce risk by 42.5%.

**Abbreviations**
ASR: age‐standardized rates
BMI: body mass index
EC: endometrial cancer
EPIC: European Prospective Investigation into Cancer and Nutrition
HT: hormone therapy
OC: oral contraceptive
RR: relative risk
WHO: World Health Organization

## Introduction

Endometrial cancer (EC) incidence rates show wide variation worldwide, with estimated age‐standardized rates (ASR; standardized to World Health Organization (WHO) World Standard Population) of 15 per 100,000 women and higher in 2018 in Europe and North America but much lower rates reported by relatively high‐quality cancer registries in parts of Africa and Asia, for example, in Algeria (ASR = 2.2/100,000) or India (ASR = 1.9/100,000).^1^ For the most part, this variation may be caused by differences in the prevalence of nongenetic and thus theoretically modifiable risk factors. Established risk factors for EC include older age, overweight and obesity, nulliparity/low parity, a relatively young age at last full‐term pregnancy, and having experienced a relatively early menarche and/or late menopause, reflecting a larger lifetime cumulative number of ovulatory menstrual cycles.^2^ In addition, the use of postmenopausal hormone therapy (HT) can either increase or decrease risk, depending on both on its composition (estrogen‐only, or estrogen‐plus‐progestin combinations)^3^ and a woman's degree of adiposity (i.e., high body mass index [BMI]).^4^ Finally, long‐term use of oral contraceptives (OC) is associated with marked reductions in EC risk, which persist for years after cessation of use,^5^ and smoking has also been associated with lower risk.^2^

Based on these established risk (and protective) factors, we previously derived a statistical model to predict a woman's absolute EC risk, in view of identifying high‐risk women who may benefit from targeted prevention measures (risk stratification), using data from the European Prospective Investigation into Cancer and Nutrition (EPIC) cohort.^6^ Here, we extend our analyses, applying this previously derived risk model to the EPIC data to (*i*) estimate the theoretical potential for the prevention of EC in Western Europe, or in similar higher‐risk populations, through risk factor avoidance or alterations in exposure patterns, and (*ii*) evaluate the extent to which the higher EC risk in the European population, as compared to a low‐risk country such as India, can be explained by the prevalence of exposure to primary risk factors.

## Methods

### EPIC cohort

The EPIC cohort was established between 1992 and 2000 at 23 centers in 10 countries: Denmark, France, Germany, Greece, Italy, the Netherlands, Norway, Spain, Sweden and the United Kingdom. Details of the study design have been published previously.^7^, ^8^ Briefly, more than 500,000 men and women mostly between the ages of 35–75 years of age were enrolled; participants provided detailed information on diet and lifestyle, including data on reproductive and menstrual history, hormone use and medical history. In all countries except France, Germany and Greece and the center of Naples, Italy, the prospective ascertainment of incident cancer cases was based on record linkage to cancer registries, whereas in France, Germany, Greece and Naples, Italy, a combination of active follow‐up with participants and their next‐of‐kin, and outcome verification with medical and health insurance records was used. In all countries, vital status was available from mortality registries. End of follow‐up for cancer outcomes and mortality for France, Germany, Greece and Naples, Italy, was the earliest of date of last contact, cancer diagnosis or death (2008–2013). For the remaining study centers included in our study, end of follow‐up ranged from 2009 (Varese and Murcia, Spain) to 2013 (San Sebastian and Asturias, Spain and Turin, Italy and Greece). Participants from Norway and Sweden were excluded due to missing data on key parameters. Ethical approval for the EPIC study was obtained from the International Agency for Research on Cancer (IARC) ethics committee, and the ethics committees of the participating centers. Participants provided informed consent.

### Statistical model

The analyses for this article are based on the EC risk prediction model we developed previously within EPIC.^6^ Briefly, this model estimates absolute risk of EC through combining a relative risk score from individual risk factor information RR(x) with age‐specific piecewise constant baseline risk, and additionally correcting for competing risk of hysterectomy or death. All age‐specific risk components are adjusted for country. The relative risk score RR(x) incorporates data on body mass index (BMI [kg/m^2^], continuous), age at menarche (per year, continuous), duration of OC use, OC use and BMI interaction (ever OC use by BMI categories; <25, 25 to <30, 30+), parity (nulliparous, 1,2,3+), age at first term pregnancy (continuous), menopausal status at recruitment (pre‐, peri‐, postmenopausal), age at menopause (per year, continuous, centered at age 50), duration of postmenopausal HT use (per year, continuous), and a smoking status and menopausal status interaction (current or former smoker by menopausal status: pre‐, peri‐, postmenopausal), and is defined as the following:

(1) $$\mathrm{RR}=\exp\;\left[0.030\times \mathrm{BMI}–0.023\times \left(\mathrm{age}\;\mathrm{at}\;\text{1st period}–13\right)–0.019\;\left(\text{if lean}\;\mathrm{OC}-\text{user}\right)–0.013\;\left(\text{if overweight}\;\mathrm{OC}-\text{user}\right)–0.036\;\left(\text{if obese}\;\mathrm{OC}-\text{user}\right)–0.023\times \text{duration of}\;\mathrm{OC}-\mathrm{use}\;\left(\text{in years}\right)–0.051\;\left(\text{if single parous}\right)–0.10\;\left(\text{if}\;2\;\text{full}-\text{term pregnancies}\right)–0.22\;\left(\text{if}\;3\;\text{or more full}-\text{term pregnancies}\right)–0.017\times \left(\mathrm{age}\;\mathrm{at}\;\text{1st full}-\text{term pregnancy}–24\right)–0.088\;\left(\text{if peri}-\text{menopausal}\right)–0.20\;\left(\text{if postmenopausal}\right)+0.029\times \left(\mathrm{age}\;\mathrm{at}\;\text{menopause}–50\right)+0.031\times \text{duration of}\;\mathrm{HT}-\mathrm{use}\;\left(\text{in years}\right)–0.11\;\left(\text{if premenopausal former smoker}\right)+0.040\;\left(\text{if premenopausal current smoker}\right)–0.12\;\left(\text{if postmenopausal former smoker}\right)–0.21\;\left(\text{if postmenopausal current smoker}\right)\;–0.14\;\left(\text{if peri}-\text{menopausal former smoker}\right)\right]$$

A more detailed description of model development and validation is given in our previous publication.^6^

### Analytic cohort and exclusions

Ten‐year risk was evaluated in our study. The analytic cohort included all women who were either diagnosed with EC within the first 10 years of follow‐up, were diagnosed with a different cancer or died during that time, or who were followed for at least 10 years (*n* = 17,467 with <10 years follow‐up excluded). Women reporting hysterectomy or prevalent cancer (except nonmelanoma skin cancer) at baseline or with no follow‐up data were excluded (*n* = 65,808). To avoid bias from extreme risk estimates for BMI in the current analysis, all women with height <130 cm (*n* = 15) and women with a BMI above 50 (*n* = 108) were excluded from our analyses. Missing values in parity (4.8%), age at first term pregnancy (0.5%), number of children (8.2%), pill use and duration of pill use (3.2%; 14.0%), HT use and duration of HT use (7.1%; 24.3%), and smoking status (2.7%) were fivefold imputed with multiple chained‐equation imputation using the R‐package “mice”.^9^, ^10^ Data on country, study center, BMI, age at recruitment, menopausal status and incident diagnosis of EC were included in the imputation models. As the risk model was originally fitted for women within the age range of 40–70 years at recruitment, risk estimates were not calculated for women younger than 40 years (*n* = 34,666) or older than 70 years (*n* = 4,630). The data set for analysis included a total of 192,089 women.

### Model calibration in the analytic cohort

Given that the analytic cohort included in our study was not identical to that included in the model development, we evaluated calibration of the model by calculating the ratio of expected to observed cases and with a calibration plot of observed *versus* expected by decile of predicted risk.

### Estimation of prevention potential

To estimate the theoretical potential for EC prevention, the risk model was used to calculate predicted 10‐year incidence rates within the cohort, with risk factors as originally observed (reference level), or under theoretical scenarios of truncated risk factor distributions, where women's exposures were truncated at theoretically feasible low‐risk levels, assigning the limit value (i.e., truncation point) for the low‐risk category to all women who had observed values outside the low‐risk range (e.g., in analyses of BMI, where 23 kg/m^2^ was defined as the truncation point, all women with BMI > 23 kg/m^2^ were assigned a value of BMI = 23 kg/m^2^). BMI was also modeled with all women with BMI > 25 kg/m^2^ with a 2.5 kg/m^2^ reduction in BMI (e.g., a 6.5 kg weight loss in a 1.61 m tall woman [corresponding to average height in EPIC]). Predicted incidence rates on the basis of these modified, hypothetical risk profiles were calculated for risk factors individually and in combination, and were examined for subsets of factors considered potentially modifiable (BMI, OC use, HT use), or not readily modifiable (ages at menarche and menopause, number of term pregnancies, age at first pregnancy) for prevention purposes.

To explore risk contours for combinations of EC risk factors, we used the relative risk model component (1) described above and defined deciles of the relative risk score using subsets of the risk factors included in the risk model: (*i*) BMI, OC use, HT use, parity, age at first birth and ages at menarche and menopause (full risk model excluding smoking); (*ii*) BMI, OC use, HT use (modifiable components of the risk model). We further evaluated deciles of the risk score using these subsets of variables, but excluding OC use given that while OC use is modifiable, it must be considered in a broader context of risks and benefits. Using the complete absolute risk model and the women's observed risk factor combinations we estimated incidence rates (i.e., predicted cases per 100,000 person‐years of follow‐up) for women in the lowest deciles of risk based on the previously defined sets of risk factors.

For comparability with EC incidence rates reported in Globocan^1^ we calculated age‐specific rates, overall and for lower‐risk profiles, for women in the EPIC cohort in 5‐year age categories. The age‐specific rates were calculated as a sum of women's predicted EC risk contributions to up to three successive 5‐year age categories, weighted by the observation times (person‐years) that women spent in each 5‐year age category during their cumulative follow‐up time of up to 10 years. We further used incidence rates for Chennai, India, between 1994 and 2008 (corresponding to the years of follow‐up in the EPIC cohort) as a representative example of incidence rates in India, a country with low incidence rates and high‐quality data from cancer registries, as age‐specific incidence rates were not available for India as a whole in Globocan 2018. Summary data for India, Europe (EU28 index), and for countries classified as having low human development index (HDI) as reported in Globocan 2012^11^ were evaluated in a secondary analysis (age‐specific incidence rates for these populations not available in Globocan 2018).

All statistical analyses were conducted with SAS 9.4 and with R (version 3.3.1, package “mice”).^9^, ^10^

### Data availability

Data are available by application to the EPIC Steering Committee (https://epic.iarc.fr/access/).

## Results

Baseline characteristics of the study population are provided in Table 1. The median age at recruitment was 52.5 years (95% range, 41–67), and median BMI was 24.3 kg/m^2^ (95% range, 18.8–36.2). At baseline, 30% of women were premenopausal, 57% had reported ever use of OCs (median duration: 5 years), and 25% had reported ever use of HT (median duration: 2 years). A total of 85% of women were parous, with 34% of women reporting 3 or more children. The median age at menarche was 13 years (95% range, 10–16) and median age at menopause was 50 (95% range, 40–57). Baseline characteristics of participants by country are provided in Supporting Information Table S1.

**Table 1:** Distribution of endometrial cancer risk factors in the analytic cohort: EPIC cohort (*n* = 192,089)

Characteristic	*n* (%) or Median (95% range)
Noncases	191,253 (100%)
Incident endometrial case in first 5 years of follow‐up	
Observed	836 (0%)
Predicted	832 (0%)
10‐year risk of EC (Median [min–max])	0.0031 (0.000; 0.186)
*n* (%)/Median (95%‐range)	
Age at recruitment	52.5 (41.1; 67.0)
Country of residence	
Denmark	24,898 (13%)
France	56,206 (29%)
Germany	12,826 (7%)
Greece	8,361 (4%)
Italy	24,874 (13%)
The Netherlands	17,679 (9%)
Spain	18,224 (9%)
United Kingdom	29,021 (15%)
Height (cm)	161 (149–174)
Weight (kg)	63.6 (47.5–93.1)
BMI (kg/m^2^)	24.3 (18.8–36.2)
Age at menarche	13 (10–16)
OC use, ever	109,196 (57%)
Duration of OC use (years)	5 (1–25)
Ever full‐term pregnancy	164,152 (85%)
One child^1^	28,872 (18%)
Two children^1^	79,775 (49%)
Three or more^1^	55,506 (34%)
Age at first full term pregnancy, years^1^	25 (18–35)
Premenopausal	56,682 (30%)
Perimenopausal	33,970 (18%)
Postmenopausal	101,437 (53%)
Age at menopause, years^2^	50 (40–57)
HT use, ever^2^	47,816 (25%)
Duration of HT use, years^3^	2.00 (0.17; 15.34)
Smoking at recruitment	
Current smoker	32,880 (17%)
Former smoker	42,732 (22%)
Never smoker	116,477 (61%)

1 Among parous women.

2 Among women postmenopausal at recruitment.

3 Among ever HT users.

Abbreviations: EC, endometrial cancer, BMI, Body mass index, OC, oral contraceptive, HT, hormone therapy.

Among the 192,089 women included in the analytic cohort, 836 were diagnosed with EC in the first 10 years of follow‐up. With risk factor profiles as observed, the risk model predicted a total of 832 incident ECs within the first 10 years, corresponding to a ratio of expected to observed cases of 1.0 (95% confidence interval, 0.93–1.06) and a predicted overall incidence rate in EPIC of 43.3/100,000 person‐years (py) of follow‐up (ASR: 11.4 per 100,000 years). A plot of observed *versus* predicted risk of EC by decile of predicted risk further documents the excellent model calibration, with observed *versus* predicted values on a straight line with slope 1.0 and zero intercept (Supporting Information Fig. S1).

We first evaluated predicted overall incidence rates for the EPIC cohort under hypothetical changes in potentially modifiable risk factors, individually and in combination (Table 2). Modeling BMI as maximally 23 kg/m^2^ or all participants with never HT use led to reductions in predicted incidence rates ranging from 10.3% (HT use, predicted incidence rate: 38.9/100,000 py) to 21.9% (BMI, predicted incidence rate 33.8/100,000 py), as compared to the cohort with risk profiles as observed; observed prevalence of BMI ≤23 kg/m^2^ and never HT use was 36% and 75%, respectively. BMI modeled with a 2.5 kg/m^2^ reduction for women with BMI > 25 kg/m^2^ resulted in a more modest 8.8% reduction in predicted incidence rates (to 39.5/100,000). Modeling BMI as maximally 23 kg/m^2^ and all participants as never HT users resulted in a 30.4% reduction in the predicted incidence rate (30.2/100,000 py; observed prevalence of both BMI ≤23 kg/m^2^ and never HT use = 27%). Assigning a minimum duration of 10 years of OC use to all women resulted in a 42.5% reduction in the predicted incidence rate (to 24.9/100,000 py; observed prevalence = 19%), whereas minimum duration of OC use of 20 years reduced the predicted incidence rate by 67.2% (to 14.2/100,000 person‐years; observed prevalence = 8%). In combination, considering the three potentially modifiable factors together and modeling BMI≤23 kg/m^2^, OC use for ≥20 years, and never use of HT for all women, the predicted incidence rate was reduced by 75.7% (to 10.5/100,000 person‐years); this combination of exposure levels, however, was observed for only 2% of the EPIC participants.

**Table 2:** Predicted reduction in crude endometrial cancer incidence rates through modification of risk factor profiles: EPIC cohort (*n* = 192,089)

	Observed prevalence of target risk profile	Cases (*n*)	Crude incidence rate (relative difference %)^1^
Observed cases over 10 years of follow‐up		836	43.5
Projected cases over 10 years		832	43.3 (ref)
Estimated predicted case numbers and incidence rates given modeled risk factor distributions			
Modeling changes in BMI and HT use			
BMI ➔ ≤23 kg/m^2^	36%	650	33.8 (−21.9%)
BMI > 25 ➔ −2.5 kg/m^2^	n/a	758	39.5 (−8.8%)
HT use ➔ never	75%	746	38.9 (−10.3%)
BMI ➔ ≤23 kg/m^2^, HT use ➔ never	27%	580	30.2 (−30.4%)
Modeling changes in OC use, alone or in combination with other risk factors			
OC use ➔ ≥10 years	19%	478	24.9 (−42.5%)
OC use ➔ ≥10 years, BMI ➔ ≤23 kg/m^2^, HT use ➔ never	6%	355	18.5 (−57.3%)
OC use ➔ ≥20 years	8%	273	14.2 (−67.2%)
OC use ➔ ≥20 years, BMI ➔ ≤23 kg/m^2^, HT use ➔ never	2%	202	10.5 (−75.7%)
Modeling changes menstrual and reproductive history			
Age at menarche ➔ ≥13 years	61%	803	41.8 (−3.5%)
Age at menopause ➔ ≤48 years	14%	741	38.6 (−11.0%)
Age 1st FTP ➔ ≥25 years	43%	784	40.8 (−5.8%)
Number of children ➔ ≥3	29%	628	32.7 (−24.6%)
Modeling changes in combinations of risk factors, other than OC			
BMI ➔ ≤23 kg/m^2^, HT use ➔ never, menarche ➔ ≥13 years, all ➔ ≥3 children	4%	420	21.8 (−49.6%)
BMI ➔ ≤23 kg/m^2^, HT use ➔ never, menarche ➔ ≥13 years, all ➔≥3 children, age 1st FTP ➔ ≥25 years, age at menopause ➔ ≤48 years	0.2%	350	18.2 (−57.9%)

1 Cases per 100,000 person‐years over 10 years of follow‐up.

We next evaluated risk factors influenced by physical and/or cultural environments, but not readily modifiable to quantify predicted reductions in EC incidence associated with differences in the prevalence of these risk factors. The risk model predicted moderate (−3.5 to −11.0%) reductions in the EC incidence rate when we modeled all EPIC women as having age at menarche ≥13 years, a first child at age ≥25 years or age at menopause ≤48 years (reported prevalence of these exposure levels ranged from 14% [age at menopause] to 61% [age at menarche]). Our analyses predicted a more substantial reduction (−24.6%; to 32.7/100,000 py) in the EC incidence rate when all women were modeled as having 3 or more children (reported by 29% of participants). The combination of low BMI (≤ 23 kg/m^2^), never use of HT, late menarche (age ≥ 13 years) and higher parity (≥3 children), a combination reported by 4% of the women in EPIC, was associated with a 49.6% reduction in the incidence rate (to 21.8/100,000 person‐years). Results were similar when the ASRs were compared, rather than the crude predicted incidence rates (Supporting Information Table S2).

Finally, we evaluated predicted incidence rates by deciles of our relative risk score, to compare these predicted rates to those reported in Globocan, specifically for lower‐risk countries. We evaluated risk profiles using the multivariable relative risk component of our model. Risk profiles were defined based on the following sets of variables: (*i*) BMI, HT use, parity, age at 1st birth, and ages at menarche and menopause (full risk model excluding smoking); and (*ii*) BMI, OC use, HT use (modifiable components of the risk model). These scores were further defined with the variables stated previously (i.e., in (*i*) and (*ii*) above) but excluding OC use. Women in the lowest decile of the relative risk score based on all variables (except smoking) had lower BMI (median 22.8 kg/m^2^), and a higher proportion were postmenopausal (68%), parous (93%; 41% with 3+ children), and reported ever use of OCs (94%; median duration 23 years), relative to the cohort overall (Supporting Information Table S3). When the scores were derived excluding OC use, ever use of OCs and OC use duration in the lowest decile were more similar to the overall cohort than for relative risk scores including OC use (e.g., score based on all factors except smoking and OC use, ever OC use = 47% (full cohort 57%) and duration = 5 years (full cohort = 5 years); variable distributions in lowest deciles of all presented relative risk scores are shown in Supporting Information Table S3).

**Figure 1:**
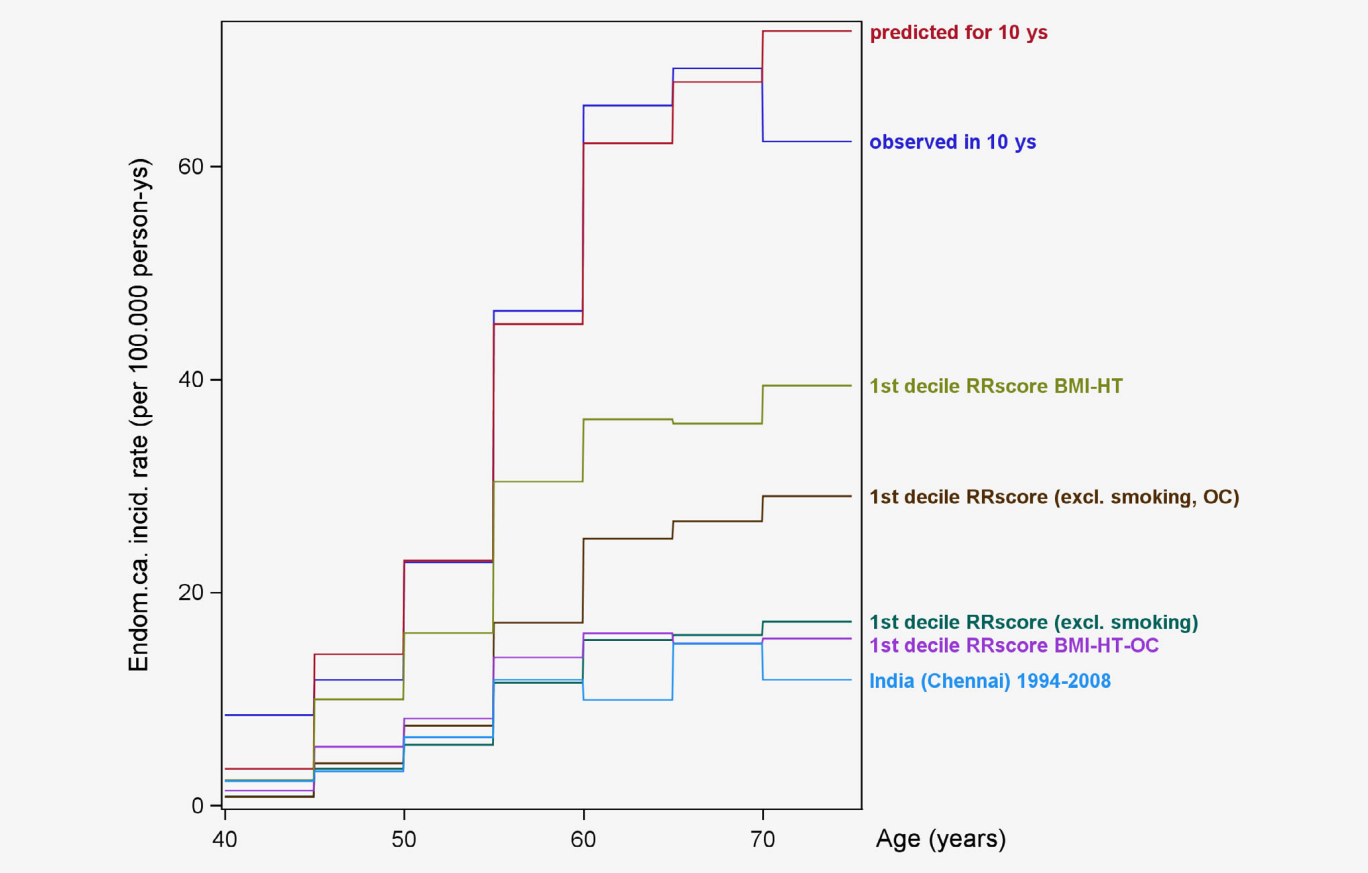
Endometrial cancer incidence, per 100,000 person years and within 5‐year categories of age, predicted in EPIC over a 10‐year follow‐up overall and for women with observed low‐risk profiles and comparison with incidence rates in India. [Color figure can be viewed at wileyonlinelibrary.com]

Figure 1 shows the age‐specific incidence rates predicted within EPIC overall, and for women in the lowest decile of the multivariable risk scores, and for India (Chennai cancer registry) as a representative “low risk” area. For women in the lowest decile of the relative risk score based on all risk factors except smoking, or based on BMI, OC use and HT use, predicted EC incidence rates were only slightly higher than those observed in India (Fig. 1). Notably, long duration of OC use in the lowest decile of risk score was particularly influential. After excluding OC use, predicted incidence rates for women in the lowest decile of the relative risk scores were substantially higher than the corresponding models including OC use. Across the 5‐year age categories, the incidence rates from the prediction model closely matched those observed in EPIC and predicted for Europe (EU28; Supporting Information Fig. S2) and the India (Chennai) data from Globocan 2018 are in line with the India country‐wide summary estimates and the low HDI estimates from Globocan 2012 (age‐specific data not available in Globocan 2018).

## Discussion

Modeling changes in exposure to high BMI and HT use, as well as to menstrual and reproductive factors, resulted in modest reductions in predicted EC incidence rates in the EPIC cohort study population. This study predicted ~30% lower EC incidence when the study population was modeled as both having relatively low BMI (≤23 kg/m^2^) and as never HT users, a combination of risk factors observed in 27% of the study population. Modest changes in the predicted incidence (≤11% reductions) were observed with modeled changes in ages at menarche and menopause, and age at first term pregnancy. Predicted incidence was 50% lower in models evaluating a low‐risk profile with BMI ≤23 kg/m^2^, never HT use, menarche at age 13 or older and 3 or more children, or 58% lower when additionally including age at first pregnancy ≥25 and age at menopause ≤48 years. These profiles as modeled are modified toward risk factor patterns observed in India in recent decades with data from India's family health surveys indicating a fertility rate of 2.9 children per woman and a low prevalence of BMI≥25 kg/m^2^ [Ref 12] (National Family Health Survey [NFHS]‐2, 1998–1999; ever‐married women aged 15–49),^12^ and national surveys reporting mean age at menarche of 14 (women born prior to 1955–1964)^13^ and mean age at menopause 47.5 years (birth cohort not specified).^14^ However, in our study population, the predicted ASR with these lower‐risk profiles (5.8 per 100,000 py and 4.8 per 100,000 py, respectively) were still >2.5‐fold higher than rates observed in India (1.9 per 100,000 in 2018), and these combinations of risk factors were observed in small proportions of EPIC participants (4 and 0.2%, respectively). We selected India as a representative lower‐risk country, given relatively high‐quality registry data (i.e., incidence rates from local/regional registries).^15^

Longer duration of OC use had a strong influence in decreasing predicted EC incidence in our study population, as illustrated by the risk patterns observed when classifying women in deciles of multivariable relative risk score based different sets of modifiable and nonmodifiable risk factors, and both including and excluding OC use. When the deciles were defined using a multivariable risk score including OC use, the lowest risk decile (i.e., 10%) of the cohort had predicted EC risk similar to that observed in a low‐risk country such as India, or in countries classified as “low” on the human development index. More moderate reductions in predicted incidence rates were observed in models in which exposure to OC use was not modified. Oral contraceptive use is less frequent in India (8.4% reported ever use, married women ages 18–49 years, 1989–1999 NFHS‐2),^12^ than in our population (ever use: 57%). Thus, while we identified a subgroup of our study population with low EC risk comparable to a low‐risk country, the relatively low risks observed in these two populations are due to different constellations of risk factors.

BMI, HT use and OC use were the three potentially modifiable risk factors we evaluated toward understanding the EC prevention potential with lower levels of exposure to EC risk factors. Higher adiposity is associated with an extensive array of sequelae, and contemporary evidence on HT use suggests limiting its use to shorter‐term therapy, in the nearer‐term following the onset of menopause for alleviation of vasomotor symptoms or in women at high risk for bone loss.^16^ Thus, the findings from our study predicting almost a third fewer EC cases in the EPIC cohort through avoiding excess body fat and abstention from HT use are in agreement with more globally accepted recommendations for exposure to these risk factors. However, it should be noted that we evaluated the prevention potential of maintaining a lower BMI in the current study, but were unable to assess the relative impact of weight change on EC risk. The strong protective effect observed with longer durations of OC use is more complicated to interpret in terms of prevention potential. While OC use is inversely associated with cancers of the endometrium,^2^ ovary,^17^, ^18^ and colorectum,^19^, ^20^ this must be balanced against higher risks of breast^21^, ^22^ and cervical cancers,^23^ and the increased risk of cerebrovascular events, together with the consideration of the contraceptive method(s) that best fit a woman's reproductive planning requirements.

While our study had important strengths, including the application of a validated risk model to evaluate the theoretical reduction in EC risk due to lifestyle modification in a large cohort, the results of our study must be considered in the context of several limitations. First, exposure data were available only from the baseline questionnaire, and we were unable to account for changes in women's exposure profiles during prospective follow‐up (e.g., women who had further pregnancies, or initiated HT use), and data on HT formulation were not available. This would result in a nondifferential misclassification of the evaluated exposures, and attenuated associations. Furthermore, we modeled EC as a composite outcome given limited data on histologic subtype. Second, the oldest women in the EPIC cohort were born in the late 1920s, and the OC pill was introduced in the 1960s. Thus, the oldest EPIC participants had lower likelihood of exposure to OCs, and would have been exposed for shorter durations, relative to women younger at recruitment. Furthermore, a birth‐cohort effect may be evident for other EC risk factors given trends toward younger age at menarche and older age at menopause, and differences in HT prescribing patterns in recent decades. Our results should be considered in the context of these secular trends in prevalence of exposure to these EC risk factors. It should also be noted that our modeled risk profiles (e.g., all women BMI ≤23 kg/m^2^) are optimistic assumptions, and so may present the upper bound of risk reduction. Finally, we compared predicted EC incidence rates in our cohort across countries and subgroups (e.g., “low HDI”) on the basis of modeled changes in risk factor distributions, and without considering the constellation of societal or infrastructure or healthcare‐related factors which also impact health outcomes.

Using a relative risk score including BMI, and HT and OC use, approximately 10% of our study population had EC risk similar to low‐risk countries. Taken together, the results of our study show that while a subset of our population had lower EC risk, on par with lower‐risk countries, we have not fully identified natural risk factors accounting for the higher EC risk in Europe. Avoidance of overweight and HT use were identified as factors that can be modified toward reducing EC risk, and lower risk of EC is an additional benefit gained from avoiding these exposures. Future studies should evaluate the prevention potential of weight loss, which we were unable to address in the current study. While relatively long duration of OC use resulted in lower predicted EC incidence, further studies evaluating a breadth of risks and benefits (including but not limited to effective contraception, but also longer‐term safety) associated with OC use are required, in particular, those evaluating more contemporary formulations, to inform populations for whom longer‐term use may be warranted for chemoprevention.

## Conflict of interest

The authors declare no conflicts of interest.

## Disclaimer

The authors alone are responsible for the views expressed in this article and they do not necessarily represent the views, decisions or policies of the institutions with which they are affiliated. Where authors are identified as personnel of the International Agency for Research on Cancer/World Health Organization, the authors alone are responsible for the views expressed in this article and they do not necessarily represent the decisions, policy or views of the International Agency for Research on Cancer/World Health Organization.

## Supporting information


**Appendix S1** Supporting information
