# Supplementary material for: Theoretical potential for endometrial cancer prevention through primary risk factor modification: Estimates from the EPIC cohort
Source: Int J Cancer. 2020 Feb 18;147(5):1325–33. doi: 10.1002/ijc.32901 (PMC8611794; doi:10.1002/ijc.32901)
Supplement: Supplementary file 1 — Appendix S1 Supporting information [file IJC-147-1325-s001.pdf]

Theoretical potential for endometrial cancer prevention through primary risk factor modification: Estimates from the EPIC cohort

Renée Turzanski Fortner, Anika Hüsing, Laure Dossus, Anne Tjønneland, Kim Overvad, Christina C. Dahm, Patrick Arveux, Agnès Fournier, Marina Kvaskoff, Matthias B. Schulze, Manuela Bergmann, Antonia Trichopoulou, Anna Karakatsani, Carlo La Vecchia, Giovanna Masala, Valeria Pala, Amalia Mattiello, Rosario Tumino, Fulvio Ricceri, Carla H. van Gils, Evelyn M. Monninkhof, Catalina Bonet, J. Ramón Quirós, Maria-Jose Sanchez, Daniel-Ángel Rodríguez-Palacios, Aurelio Barricarte, Pilar Amiano, Naomi E. Allen, Ruth C. Travis, Marc J. Gunte[2], Vivian Viallon, Elisabete Weiderpass, Elio Riboli, Rudolf Kaaks

Table of contents

1) **Supplementary Figure 1**: Calibration of 10-year EC-risk estimates in the EPIC cohort
2) **Supplementary Figure 2**: Endometrial cancer incidence, per 100,000 person years and within 5-year categories of age, observed, and predicted in EPIC over a 10-year follow-up and comparison with incidence rates for Europe (EU28) and countries with low human development index, India and the India (Chennai) registry
3) **Supplemental Table 1:** Distribution of endometrial cancer risk factors by country: EPIC cohort (n=192,089)
4) **Supplemental Table 2**: Predicted reduction in endometrial cancer age standardized incidence rates (ASRs) through modification of risk factor profiles: EPIC cohort (n=192,089)
5) **Supplemental Table 3:** Distribution of endometrial cancer risk factors overall, and for women in the lowest decile of selected, multi-variable risk-scores: EPIC cohort (n=192,089)

Supplementary Figure 1: **Calibration of 10-year EC-risk estimates in the EPIC cohort**

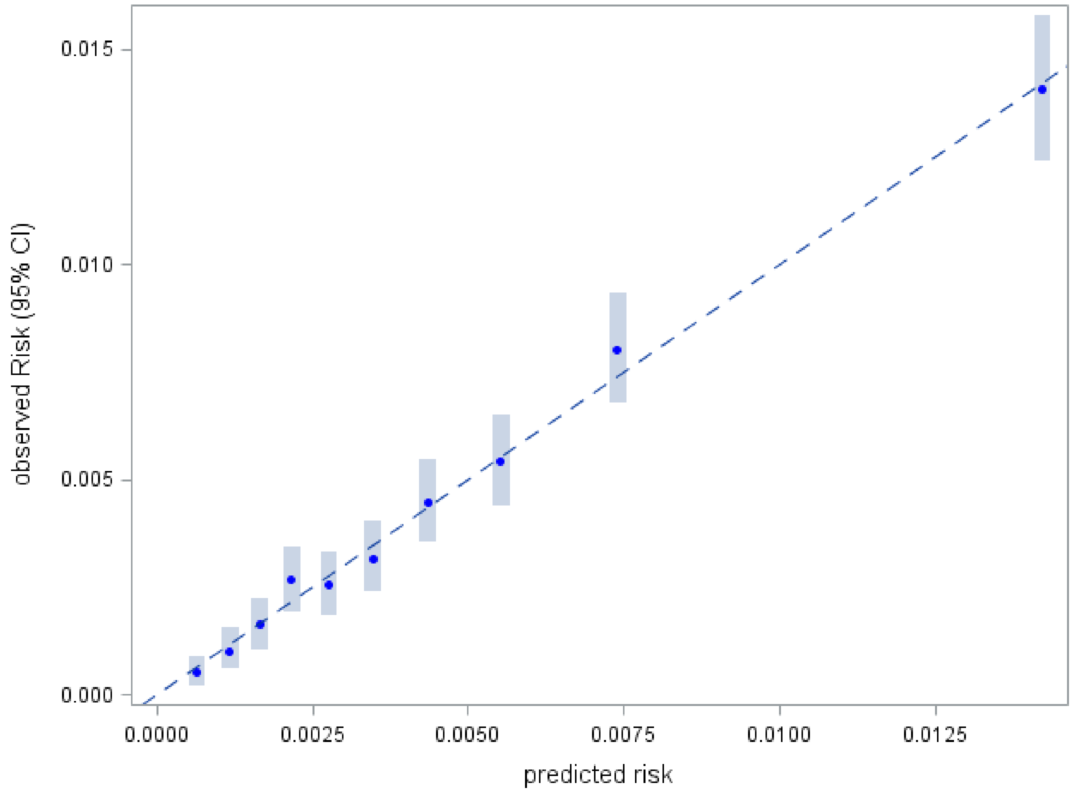

Supplementary Figure 2:

Endometrial cancer incidence, per 100,000 person years and within 5-year categories of age, observed, and predicted in EPIC over a 10-year follow-up and comparison with incidence rates for Europe (EU28) and countries with low human development index, India and the India (Chennai) registry

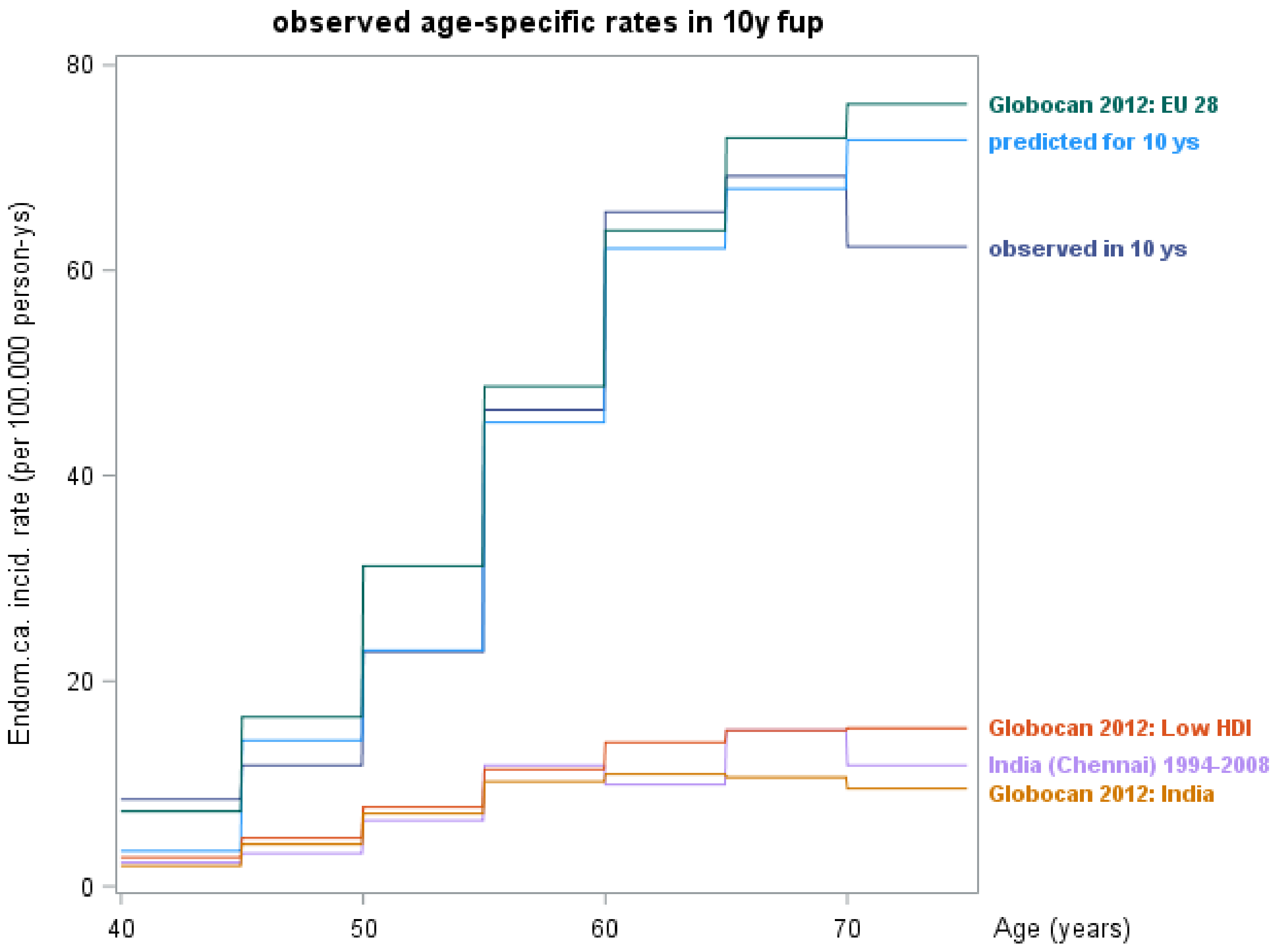

Supplemental Table 1: Distribution of endometrial cancer risk factors by country: EPIC cohort (n=192,089)

N (%) or Median (95%-range)		France	Italy	Spain	United Kingdom	The Netherlands	Greece	Germany	Denmark
All		56206	24874	18224	29021	17679	8361	12826	24898
Incident EC		167 (0%)	116 (0%)	76 (0%)	134 (0%)	82 (0%)	19 (0%)	72 (1%)	170 (1%)
decile RR-score:	1	4765 (8%)	960 (4%)	350 (2%)	3012 (10%)	3866 (22%)	119 (1%)	2099 (16%)	4038 (16%)
	2	5820 (10%)	1524 (6%)	582 (3%)	3236 (11%)	2775 (16%)	316 (4%)	1866 (15%)	3092 (12%)
	3	6541 (12%)	1850 (7%)	851 (5%)	3326 (11%)	2219 (13%)	400 (5%)	1373 (11%)	2647 (11%)
	4	6926 (12%)	2052 (8%)	1094 (6%)	3146 (11%)	1902 (11%)	471 (6%)	1129 (9%)	2489 (10%)
	5	6914 (12%)	2269 (9%)	1338 (7%)	3232 (11%)	1523 (9%)	537 (6%)	1134 (9%)	2262 (9%)
	6	6665 (12%)	2694 (11%)	1594 (9%)	3100 (11%)	1367 (8%)	640 (8%)	1042 (8%)	2106 (8%)
	7	6257 (11%)	3036 (12%)	2006 (11%)	2878 (10%)	1184 (7%)	802 (10%)	1028 (8%)	2018 (8%)
	8	5544 (10%)	3441 (14%)	2522 (14%)	2655 (9%)	1067 (6%)	1067 (13%)	987 (8%)	1925 (8%)
	9	4331 (8%)	3648 (15%)	3370 (18%)	2422 (8%)	950 (5%)	1596 (19%)	991 (8%)	1902 (8%)
	10	2443 (4%)	3399 (14%)	4518 (25%)	2014 (7%)	827 (5%)	2413 (29%)	1177 (9%)	2419 (10%)
Age		50.95 (43.60 - 66.31)	51.71 (40.73 - 64.68)	49.77 (40.46 - 64.27)	51.58 (40.70 - 68.72)	54.37 (41.82 - 68.65)	55.79 (40.77 - 69.20)	51.34 (40.53 - 64.56)	56.16 (50.52 - 64.74)
Body height [cm]		161 (150 - 173)	158 (147 - 171)	156 (146 - 168)	162 (151 - 174)	164 (152 - 177)	156 (144 - 168)	163 (151 - 175)	164 (153 - 176)
Body weight [kg]		59 (45 - 83)	63 (48 - 91)	68 (53 - 96)	64 (48 - 94)	67 (50 - 96)	69 (51 - 99)	66 (50 - 99)	67 (50 - 98)
Body mass index [kg/m²]		22.3 (18.1 - 31.2)	25.1 (19.4 - 36.2)	28.1 (21.4 - 39.4)	24.0 (18.8 - 35.5)	24.7 (19.2 - 35.4)	28.7 (21.1 - 40.6)	24.8 (19.2 - 37.2)	24.7 (19.2 - 36.1)
Age at menarche		13 (10 - 16)	13 (10 - 16)	13(10 - 16)	13 (10 - 16)	13 (11 - 17)	13 (11 - 17)	13(10 - 17)	14 (11 - 17)
Ever ues of OC		35791 (64%)	9842 (40%)	6620 (36%)	18899 (65%)	12429 (70%)	632 (8%)	10245 (80%)	14738 (59%)
Duration of OC-use [years]		5 (1 - 25)	2 (1 - 22)	2 (1 - 14)	6 (1 - 25)	10 (1 - 25)	1 (1 - 12)	10 (1 - 25)	6 (1 - 25)
Ever full term pregnancy		47789 (85%)	21584 (87%)	16328 (90%)	23103 (80%)	14922 (84%)	7609 (91%)	10841 (85%)	21976 (88%)
# children:	1	8601 (18%)	5392 (25%)	1474 (9%)	3722 (16%)	1664 (11%)	833 (11%)	3291 (30%)	3894 (18%)
	2	23703 (50%)	10801 (50%)	6172 (38%)	11544 (50%)	6705 (45%)	4207 (55%)	5304 (49%)	11338 (52%)
	3 or more	15485 (32%)	5391 (25%)	8681 (53%)	7837 (34%)	6553 (44%)	2569 (34%)	2246 (21%)	6744 (31%)
Age at first full term pregnancy		24 (19 - 35)	25 (19 - 36)	25 (19 - 34)	25 (18 - 37)	25 (18 - 36)	24 (17 - 36)	24 (18 - 36)	23 (17 - 34)

Pre menopause	16287 (29%)	8671 (35%)	8418 (46%)	10216 (35%)	3868 (22%)	2186 (26%)	4931 (38%)	2105 (8%)
Post menopause	24541 (44%)	12149 (49%)	7853 (43%)	14926 (51%)	10709 (61%)	5414 (65%)	6287 (49%)	19558 (79%)
Peri-menopausal	15378 (27%)	4054 (16%)	1953 (11%)	3879 (13%)	3102 (18%)	761 (9%)	1608 (13%)	3235 (13%)
Age at menopause	51 (40 - 57)	50 (40 - 56)	50 (38 - 56)	50 (39 - 57)	50 (40 - 56)	49 (37 - 56)	50 (45 - 58)	50 (40 - 57)
Ever use of HT	16157 (29%)	3976 (16%)	1660 (9%)	7438 (26%)	3467 (20%)	415 (5%)	4527 (35%)	10176 (41%)
Duration of HT-use [years]	2.1 (0.1 - 10.8)	1.0 (0.5 - 8.0)	1.0 (0.5 - 6.0)	2.0 (0.1 - 10.4)	2.0 (0.5 - 12.0)	0.7 (0.1 - 10.0)	3.0 (0.5 - 13.0)	4.0 (0.5 - 20.0)
Never smoked	39637 (71%)	13447 (54%)	14253 (78%)	16943 (58%)	7123 (40%)	6817 (82%)	7290 (57%)	10966 (44%)
Former smoker	11455 (20%)	4983 (20%)	1446 (8%)	8941 (31%)	5974 (34%)	440 (5%)	3361 (26%)	6132 (25%)
Current smoker	5114 (9%)	6444 (26%)	2526 (14%)	3136 (11%)	4582 (26%)	1103 (13%)	2175 (17%)	7799 (31%)
10-year risk of developing EC	0.30% (0.01-7.91%)	0.35% (0.00-7.18%)	0.31% (0.01-8.41%)	0.29% (0.00-8.11%)	0.36% (0.00-8.46%)	0.12% (0.00-1.72%)	0.25% (0.01-9.88%)	0.41% (0.02-18.56%)
10-year risk for a woman with fixed average risk profile:								
Median EPIC*	0.34%	0.30%	0.24%	0.33%	0.44%	0.07%	0.39%	0.34%

*) Median EPIC has the overall average risk profile: age=53 years, menopausal status=postmenopausal since age 50 years, HT=never users, full-term births=2, age at first term birth=25 years, menarche at age=13 years, OC pill use=5 years, BMI=24.3, smoker= never

Supplemental Table 2: Predicted reduction in endometrial cancer age standardized incidence rates (ASRs) through modification of risk factor profiles: EPIC cohort (n=192,089)

	Observed prevalence of target risk profile	Cases (n)	Age Standardized Incidence Rate (relative difference %)*
Observed cases over 10 years of follow-up		836	11.3
Projected cases over 10 years		832	11.4
**Estimated predicted case numbers and incidence rates given modeled risk factor distributions**			
**Modeling changes in BMI and HT use**			
BMI → ≤ 23 kg/m^2	36%	650	8.9 (-22.1%)
BMI>25 → -2.5 kg/m^2	n/a	758	10.4 (-9.1%)
HT use → never	75%	746	10.3 (-9.8%)
BMI → ≤ 23 kg/m^2, HT use → never	27%	580	7.9 (-30.1%)
**Modeling changes in OC use, alone or in combination with other risk factors**			
OC use → ≥ 10 years	19%	478	6.5 (-43.2%)
OC use → ≥ 10 years, BMI → ≤ 23 kg/m^2, HT use → never	6%	355	4.8 (-57.5%)
OC use → ≥ 20 years	8%	273	3.7 (-67.7%)
OC use → ≥ 20 years, BMI → ≤ 23 kg/m^2, HT use → never	2%	202	2.75 (-75.9%)
**Modeling changes menstrual and reproductive history**			
Age at menarche → ≥ 13 years	61%	803	11.0 (-3.5%)
Age at menopause → ≤ 48 years	14%	741	10.1 (-11.7%)
Age 1st FTP → ≥ 25 years	43%	784	10.7 (-5.6%)
Nr. of children → ≥ 3	29%	628	8.6 (-24.6%)
**Modeling changes in combinations of risk factors, other than OC**			
BMI → ≤ 23 kg/m^2, HT use → never, menarche → ≥ 13 years, all → ≥ 3 children	4%	420	5.8 (-49.5%)
BMI → ≤ 23 kg/m^2, HT use → never, menarche → ≥ 13 years, all → ≥ 3 children, age 1st FTP → ≥ 25 years, age at menopause → ≤48	0.2%	350	4.8 (-58.1%)

* cases per 100,000 person-years over 10 years of follow-up, standardized to the WHO World Standard Population.

Supplemental Table 3: Distribution of endometrial cancer risk factors overall, and for women in the lowest decile of selected, multi-variable risk-scores: EPIC cohort (n=192,089)

N (%) / Median (95%-range)	Cohort (N=192,089)	0-10% RR-score Based on all factors except smoking	0-10% RR-score Based on all factors except smoking and OC	0-10% RR-score Based on HT use, OC use, and BMI	0-10% RR-score Based on HT use and BMI
Non cases	191253 (100%)				
Incident endometrial case in first 10 years of follow-up					
Observed	836 (0%)	22 (0%)	33 (0%)	20 (0%)	32 (0%)
Predicted	832 (0%)	22 (0%)	41 (0%)	21 (0%)	42 (0%)
10-year risk of EC (Median (min - max))					
	0.0031 (0.000;0.186)	0.0010(0.00;0.0036)	0.002 (0.000;0.0073)	0.0009 (0.0000;0.0089)	0.0018 (0.000;0.0143)
			N (%) or Median (95% range)		
Age at recruitment	52.53 (41; 67)	53.5 (42.1; 66.6)	57.5 (45.2; 68.5)	50.2 (40.9; 64.2)	48.4 (40.9; 65.6)
Country of residence					
Denmark	24898 (13%)	3624 (19%)	2865 (15%)	2889 (15%)	1401 (7%)
France	56206 (29%)	5321 (28%)	4778 (25%)	6535 (34%)	10847 (56%)
Germany	12826 (7%)	2244 (12%)	568 (3%)	3159 (16%)	878 (5%)
Greece	8361 (4%)	171 (1%)	1033 (5%)	9 (0%)	141 (1%)
Italy	24874 (13%)	759 (4%)	2409 (13%)	530 (3%)	1613 (8%)
Netherlands	17679 (9%)	3615 (19%)	2971 (15%)	3060 (16%)	1288 (7%)
Spain	18224 (9%)	404 (2%)	1531 (8%)	120 (1%)	205 (1%)
United Kingdom	29021 (15%)	3071 (16%)	3055 (16%)	2907 (15%)	2836 (15%)
Height (cm)	161 (149 - 174)	163.0 (150.5;175.0)	161.0 (148.0;174.0)	163.00 (151.8;175.0)	162.4 (151.0;175.0)
Weight (kg)	63.6 (47.5 - 93.1)	60.8 (45.6; 81.5)	59.0 (44.5; 78.0)	60.36 (45.9; 81.4)	51.3 (42.0; 60.4)
Body mass index (BMI; kg/m^2)	24.3 (18.8 - 36.2)	22.8 (18.0; 29.7)	22.6 (17.6; 29.7)	22.60 (18.0; 29.8)	19.6 (17.0; 20.6)
Age at menarche	13 (10 - 16)	13 (11; 17)	14 (11; 17)	13 (10; 16)	13 (11; 16)
OC use, ever	109196 (57%)	17986 (94%)	9043 (47%)	19209 (100%)	13208 (69%)
Duration of OC use (years)	5 (1 - 25)	23 (1; 25)	5 (1; 25)	24 (10; 25)	5 (1; 25)
Ever full-term pregnancy	164152 (85%)	17933 (93%)	18455 (96%)	16377 (85%)	15495 (81%)
1 child*	28872 (18%)	2801 (16%)	2551 (14%)	3340 (20%)	3521 (23%)
2 children*	79775 (49%)	7695 (43%)	5777 (31%)	8698 (53%)	8041 (52%)
3 or more*	55506 (34%)	7436 (41%)	10127 (55%)	4339 (26%)	3933 (25%)
Age at first full term pregnancy, years*	25 (18 - 35)	25 (19; 38)	27 (20; 39)	24 (18; 36)	25 (19; 36)
Premenopausal	56682 (30%)	2187 (11%)	144 (1%)	6558 (34%)	8703 (45%)
Perimenopausal	33970 (18%)	3922 (20%)	765 (4%)	5385 (28%)	4799 (25%)
Postmenopausal	101437 (53%)	13099 (68%)	18300 (95%)	7266 (38%)	5707 (30%)

Age at menopause, years†	50 (40 - 57)	48 (32; 55)	46 (34; 53)	50 (40; 57)	50 (39; 56)
HT use, ever†	47816 (25%)	5625 (29%)	4310 (22%)	4382 (23%)	1524 (8%)
Duration of HT use, years‡	2.00 (0.17; 15.34)	2 (0.17; 10)	1(0.08; 6)	2 (0.17; 8)	0.5 (0.08; 2.5)
Smoking at recruitment					
Current smoker	32880 (17%)	4213 (22%)	3759 (20%)	4034 (21%)	3601 (19%)
Former smoker	42732 (22%)	4985 (26%)	4052 (21%)	5162 (27%)	4272 (22%)
Never smoker	116477 (61%)	10011 (52%)	11398 (59%)	10014 (52%)	11336 (59%)

Abbreviations: EC=endometrial cancer; BMI=Body mass index; OC=oral contraceptive; HT=hormone therapy
*Among parous women; †Among women postmenopausal at recruitment; ‡Among ever HT users
